# Japanese encephalitis virus induces matrix metalloproteinase-9 expression via a ROS/c-Src/PDGFR/PI3K/Akt/MAPKs-dependent AP-1 pathway in rat brain astrocytes

**DOI:** 10.1186/1742-2094-9-12

**Published:** 2012-01-18

**Authors:** Chuen-Mao Yang, Chih-Chung Lin, I-Ta Lee, Yi-Hsin Lin, Caleb M Yang, Wei-June Chen, Mei-Jie Jou, Li-Der Hsiao

**Affiliations:** 1 Department of Physiology and Pharmacology, Chang Gung University, Kwei-San, Tao-Yuan, Taiwan; 2 Health Aging Research Center, Chang Gung University, Kwei-San, Tao-Yuan, Taiwan; 3 Department of Anesthetics, Chang Gung University and Chang Gung Memorial Hospital, Kwei-San, Tao-Yuan, Taiwan; 4 School of Medicine, National Yang Ming University, Taipei, Taiwan; 5 Department of Public Health and Parasitology, Chang Gung University, Kwei-San, Tao-Yuan, Taiwan

## Abstract

**Background:**

Japanese encephalitis virus (JEV) infection is a major cause of acute encephalopathy in children, which destroys central nervous system (CNS) cells, including astrocytes and neurons. Matrix metalloproteinase (MMP)-9 has been shown to degrade components of the basal lamina, leading to disruption of the blood-brain barrier (BBB) and to contribute to neuroinflammatory responses in many neurological diseases. However, the detailed mechanisms of JEV-induced MMP-9 expression in rat brain astrocytes (RBA-1 cells) are largely unclear.

**Methods:**

In this study, the effect of JEV on expression of MMP-9 was determined by gelatin zymography, western blot analysis, RT-PCR, and promoter assay. The involvement of AP-1 (c-Jun and c-Fos), c-Src, PDGFR, PI3K/Akt, and MAPKs in these responses were investigated by using the selective pharmacological inhibitors and transfection with siRNAs.

**Results:**

Here, we demonstrate that JEV induces expression of pro-form MMP-9 via ROS/c-Src/PDGFR/PI3K/Akt/MAPKs-dependent, AP-1 activation in RBA-1 cells. JEV-induced MMP-9 expression and promoter activity were inhibited by pretreatment with inhibitors of AP-1 (tanshinone), c-Src (PP1), PDGFR (AG1296), and PI3K (LY294002), and by transfection with siRNAs of c-Jun, c-Fos, PDGFR, and Akt. Moreover, JEV-stimulated AP-1 activation was inhibited by pretreatment with the inhibitors of c-Src, PDGFR, PI3K, and MAPKs.

**Conclusion:**

From these results, we conclude that JEV activates the ROS/c-Src/PDGFR/PI3K/Akt/MAPKs pathway, which in turn triggers AP-1 activation and ultimately induces MMP-9 expression in RBA-1 cells. These findings concerning JEV-induced MMP-9 expression in RBA-1 cells imply that JEV might play an important role in CNS inflammation and diseases.

## Background

Japanese encephalitis virus (JEV) is a single-stranded, positive-sense RNA virus belonging to the family flaviviridae. JEV is transmitted between animals and human host by culex mosquitoes [1,2]. After the bite of an infected mosquito, JEV amplifies peripherally producing transient viremia before entering into the central nervous system (CNS) [2]. The principal target cells for JEV are in the CNS, and include neurons and astrocytes [3]. Several lines of evidence suggest that JEV frequently causes severe encephalitic illness, and is one of the most important endemic encephalitides in the world, especially in Eastern and Southeastern Asia, clinically manifesting with fever, headache, vomiting, signs of meningeal irritation and altered consciousness leading to high mortality [1-3]. In CNS injuries and in diseases such as encephalitis, matrix metalloproteinases (MMPs) play an important role in the regulation of pathological processes in the CNS [4-6].

MMPs constitute a family of more than 25 enzymes, which process a large number of pericellular substrates. The distinctive characteristics of this subgroup of matrixins is their dependence on zinc ion at the active site, the presence of a cysteine switch motif in the propeptide, and a zinc-binding domain in the catalytic domain [7]. In the CNS, MMPs are implicated in various processes involved in development, such as migration of precursor cells, axonal outgrowth, and myelinogenesis. In accordance with the role of MMPs in degrading the extracellular microenvironment, gelatinases might regulate the migration of different neural cell types to their final destinations [7]. In addition, MMPs also regulate CNS pathological processes that may contribute to the progression of CNS injuries and diseases, such as demyelination, blood-brain barrier (BBB) and blood-nerve barrier opening, invasion of neural tissue by blood-derived immune cells, modulation of neuroinflammation, and direct neurotoxicity [6,8,9]. within the MMP family, gelatinases, MMP-2 and MMP-9 mediate lesion development in response to brain injury. MMP-2 (gelatinase A; 72 kDa) is constitutively expressed by several cell types, including brain cells. In contrast, basal levels of MMP-9 (gelatinase B; 92 kDa) are usually low in normal physiological conditions and are increased by various stimuli, such as TNF-α and IL-1β [6,10-12].

Up-regulation of MMP-9 by viral infection has been shown to trigger tissue injury in various organs. For instance, the gp120 protein of the human immunodeficiency virus (HIV) disrupts the BBB by increasing MMP-9 and reducing vascular tight junction proteins via mechanisms involving ROS generation and oxidant injury [13,14]. Moreover, our previous study demonstrated that JEV induces expression of MMP-9 that causes brain damage in mice, and that this expression is reduced by pretreatment with MMP-9 inhibitor in vivo [15].

Expression of MMP-9 can be induced by extracellular stimuli at the transcriptional and translational levels [16,17]. Many reports have shown that the promoter of MMP-9 possesses a series of functional activator/enhancer element-binding sites, including NF-κB and activator protein-1 (AP-1) [5,9]. Our previous study reported that JEV-induced MMP-9 expression is mediated through NF-κB [15], but the role of AP-1 in MMP-9 gene expression induced by JEV is still unknown. AP-1 is a dimeric transcription factor comprising proteins from several families whose common denominator is possession of basic leucine zipper (bZIP) domains that are essential for dimerization and DNA binding. Moreover, various stimuli lead to the expression and/or activation of c-Fos and c-Jun products which heterodimerize and bind to AP-1 sites within MMP-9 gene promoters [18]. Recent studies have further demonstrated that several external stimuli can up-regulate MMP-9 expression via AP-1 in different cell types [19,20]. Therefore, in this study, we sought to determine whether expression of MMP-9 by JEV infection is mediated through AP-1.

Several factors can activate signaling transductions that enhance AP-1 activity [21]. For example, in NIH 3T3 mouse fibroblasts, platelet-derived growth factor (PDGF)-stimulated JNK1/2-dependent activation of c-Jun and p42/p44 MAPK-dependent activation of c-Fos leads to the expression of c-myc that regulates normal and aberrant cell growth [22,23]. In addition, iron increases MMP-9 expression by increasing AP-1 binding via p42/p44 MAPK and Akt activation in head and neck squamous carcinoma cells [24]. Moreover, several studies have shown that stimulation of the signaling pathways by viral infection, such as hepatitis B virus (HBV), influenza virus, and Kaposi's sarcoma-associated herpesvirus leads to activation of AP-1 [25,26]. Nonetheless, the mechanisms underlying JEV-stimulated activation of signaling pathways associated with AP-1 in astrocytes are not completely elucidated.

A recent study from our laboratory shows that JEV-induced MMP-9 expression is mediated through reactive oxygen species (ROS)/MAPKs-dependent NF-κB activation in rat brain astrocytes (RBA-1 cells) [15]. In the present study, the major signaling pathways linked to AP-1 activation and MMP-9 expression by JEV were investigated in RBA-1 cells. Our results demonstrate that JEV-induced MMP-9 expression is mediated through ROS/c-Src/PDGFR/PI3K/Akt/MAPKs-dependent activation of AP-1 signaling pathway in RBA-1 cells.

## Methods

### Materials

Anti-PDGFR, anti-c-Src, anti-Akt, anti-c-Fos, and anti-β-actin antibodies were obtained from Santa Cruz (Santa Cruz, CA). Anti-phospho-PDGFR, anti-phospho-c-Src, anti-phospho-p42/p44 MAPK, anti-phospho-p38 MAPK, anti-phospho-JNK1/2, and anti-phospho-Akt antibodies were from Cell Signaling (Danver, MA). Anti-GAPDH antibody was from Biogenesis (Bournemouth, UK). AG1296, PP1, LY294002, U0126, SB203580, SP600125, tanshinone, and diphenyleneiodonium chloride (DPI) were from Biomol (Plymouth Meeting, PA). Apocynin (APO) was purchased from ChromaDex (Santa Ana, CA). N-acetyl-L-cysteine (NAC), gelatin, enzymes, and other chemicals from Sigma (St. Louis, MO).

### Preparation of viruses

The T1P1 strain of JEV was propagated in C6/36 cells as previously described [15]. The titer of JEV was determined by a plaque assay.

### Rat brain astrocyte-1 culture

RBA-1 cells were used throughout this study. This cell line originated from a primary astrocyte culture of neonatal rat cerebrum and naturally developed through successive cell passages. RBA-1 cells were stained for glial fibrillary acid protein (GFAP) as an astrocyte-specific marker and used within 40 passages, which show normal cellular morphological characteristics and have steady growth and proliferation in the monolayer system [16].

### MMP gelatin zymography

After JEV treatment, culture medium was collected and mixed with equal amounts of non-reduced sample buffer and electrophoresed on 10% SDS-polyacrylamide gels containing 1 mg/ml gelatin as a protease substrate. Following electrophoresis, gels were placed in 2.7% Triton X-100 for 30 min to remove SDS, and then incubated for 24 h at 37°C in developing buffer (50 mM Tris base, 40 mM HCl, 200 mM NaCl, 5 mM CaCl_2_, and 0.2% Briji 35; Novex) on a rotary shaker. After incubation, gels were stained in 30% methanol, 10% acetic acid, and 0.5% w/v Coomassie brilliant blue for 10 min followed by destaining. Mixed human MMP-2 and MMP-9 standards (Chemicon) were used as positive controls. Gelatinolytic activity was manifested as horizontal white bands on a blue background. Since cleaved MMPs are not reliably detected, only proform zymogens were quantified.

### Transient transfection with siRNAs

The small interfering RNA (siRNA) duplexes corresponding to rat c-Fos (RSS320774, RSS320772, and RSS359279), c-Jun (RSS340670, RSS351339, and RSS340668), PDGFR (RSS351968, RSS351967, and RSS351966), Akt (RSS301983, RSS301984, and RSS301985), and scrambled siRNA were from Invitrogen (Carlsbad, CA). Transient transfection of siRNAs (100 nM) was performed using a Lipofetamine™ RNAiMAX reagent according to the manufacturer's instructions.

### Total RNA extraction and RT-PCR analysis

Total RNA was isolated from RBA-1 cells (10-cm culture dishes) incubated with JEV for the indicated time intervals, using TRIzol according to the protocol of the manufacturer. RNA concentration was spectrophotometrically determined at 260 nm. First strand cDNA synthesis was performed with 2 mg of total RNA using random hexamers as primers in a final volume of 20 ml (5 mg/ml random hexamers, 1 mM dNTPs, 2 units/ml RNasin, and 10 units/ml Moloney murine leukemia virus reverse transcriptase). The reaction was carried out at 37°C for 60 min. cDNAs encoding β-actin, MMP-9, c-Jun, and c-Fos were amplified from 3-5 ml of the cDNA reaction mixture using specific gene primers. Oligonucleotide primers for MMP-9, β-actin, c-Jun, and c-Fos were as follow:

#### MMP-9

5'-AGTTTGGTGTCGCGGAGCAC-3' (sense)

5'-TACATGAGCGCTTCCGGCAC-3' (antisense)

#### β-actin

5'-TGACGGGGTCACCCACACTGTGCCCATCTA-3' (sense)

5'-CTAGAAGCATTTGCGGTGGACGATG-3' (antisense)

#### c-Jun

5'-ATGACTGCAAAGATGGAAACG-3' (sense)

5'-TATTCTGGCTATGCAGTTCAG-3' (anti-sense)

#### c-Fos

5'-ACTGCGAGAACCAAGCTACTGCTG-3' (sense)

5'-GTACGTCCATTGACATGTTGCTCAG-3' (anti-sense)

### Western blot analysis

Growth-arrested RBA-1 cells were incubated with JEV at 37°C for various time intervals. The cells were washed with ice-cold PBS, scraped, and collected by centrifugation at 45000 × g for 1 h at 4°C to yield the whole cell extract, as previously described [16]. Samples were denatured, subjected to SDS-PAGE using a 10% (w/v) running gel, and transferred to nitrocellulose membrane. Membranes were incubated overnight using an anti-phospho-PDGFR, anti-phospho-c-Src, anti-phospho-Akt, anti-phospho-p42/p44 MAPK, anti-phospho-JNK1/2, anti-phospho-p38 MAPK, or anti-GAPDH antibody. Membranes were then washed with TTBS four times for 5 min each, incubated with 1:2000 dilution of anti-rabbit or anti-mouse horseradish peroxidase antibody for 1 h at room temperature. Immunoreactive bands were detected using ECL reagents.

### Co-immunoprecipitation assay

Cell lysates containing 1 mg of proteins were incubated with 2 μg of anti-c-Src antibody at 4°C for 1 h, and then 10 μl of 50% protein A-agarose beads was added and mixed at 4°C for 16 h. The immunoprecipitates were collected and washed thrice with lysis buffer without Triton X-100; 5× Laemmli buffer was added, and then subjected to electrophoresis on 10% SDS-PAGE. Western blot analysis was performed using an antibody against either anti-c-Src or anti-phospho-PDGFR antibody.

### Rat MMP-9 promoter cloning, transient transfection, and promoter activity assay

The upstream region (-1280 to +19) of the rat MMP-9 promoter was cloned to the pGL3-basic vector containing the luciferase reporter system. Briefly, a 1.3-kb segment at the 5'-flanking region of the rat MMP-9 gene was amplified by PCR using specific primers for the rat MMP-9 gene (accession no. U36476): 5'-ccccggtaccGAAGGCGAAATGCTTTGCCC (forward/Kpn1) and 5'-ccccctcgaGGGTGAGAACCGAAGCTTCTG (reverse/Xho1). The pGL3-Basic vector, containing a polyadenylation signal upstream from the luciferase gene, was used to construct the expression vectors by subcloning PCR-amplified DNA of the MMP-9 promoter into the Kpn1/Xho1 site of this vector. The PCR products (pGL3-MMP-9WT) were confirmed by their size, as determined by electrophoresis and by DNA sequencing. Additionally, the introduction of a mismatched primer mutation into the AP-1 to generate pGL3-MMP-9 distal ΔAP-1/wtEts was performed, using the following (forward) primer: distal ΔAP-1/wtEts: 5'-GCAGGAGAGGAAGCTGAGTTGAAGACA-3'. All plasmids were prepared by using QIAGEN plasmid DNA preparation kits. The MMP-9 promoter reporter construct was transfected into RBA-1 cells using the Lipofectamine reagent according to the instructions of the manufacturer. To assess promoter activity, cells were collected and disrupted by sonication in lysis buffer (25 mM Tris-phosphate, pH 7.8, 2 mM EDTA, 1% Triton X-100, and 10% glycerol). After centrifugation, aliquots of the supernatants were tested for luciferase activity using the luciferase assay system. Firefly luciferase activities were standardized to those of β-galactosidase activity.

### Chromatin immunoprecipitation assay

To detect the *in vivo *association of nuclear proteins with rat MMP-9 promoter, chromatin immunoprecipitation (ChIP) analysis was conducted as previously described [27]. Briefly, RBA-1 cells were cross-linked with 1% formaldehyde for 10 min at 37°C and washed thrice with ice-cold PBS containing 1 mM phenylmethylsulfonyl fluoride (PMSF) and 1% aprotinin. Soluble chromatin was prepared using a ChIP assay kit (Upstate) according to the manufacturer's recommendations and immunoprecipitated without (control) or with anti-c-Fos or anti-c-Jun antibody and normal goat immunoglobulin G (IgG). Following washes and elution, precipitates were heated overnight at 65°C to reverse cross-linking of DNA and protein. DNA fragments were purified by phenol-chloroform extraction and ethanol precipitation. The purified DNA was subjected to PCR amplification using the primers specific for the region (-597 to -318) containing the distal AP-1 binding site (-503 to -497) present in the MMP-9 promoter region, sense primer: 5'-AGAGCCTGCTCCCAGAGGGC-3'; antisense primer: 5'-GCCAAGTCAGGCAGGACCCC-3'. PCR fragments were analyzed on 2% agarose in 1× TAE gel containing ethidium bromide and the size (279 bp) was compared to a molecular weight marker.

### Statistical analysis of data

Concentration-effect curves were fitted and EC50 values were estimated using a GraphPad Prism Program (GraphPad, San Diego, CA, USA). Data were expressed as mean ∀ S.E.M. and analyzed by one-way ANOVA followed with Tukey's post-hoc test. P < 0.05 was considered significant.

## Results

### AP-1 is involved in JEV-induced proMMP-9 expression

**Figure 1:**
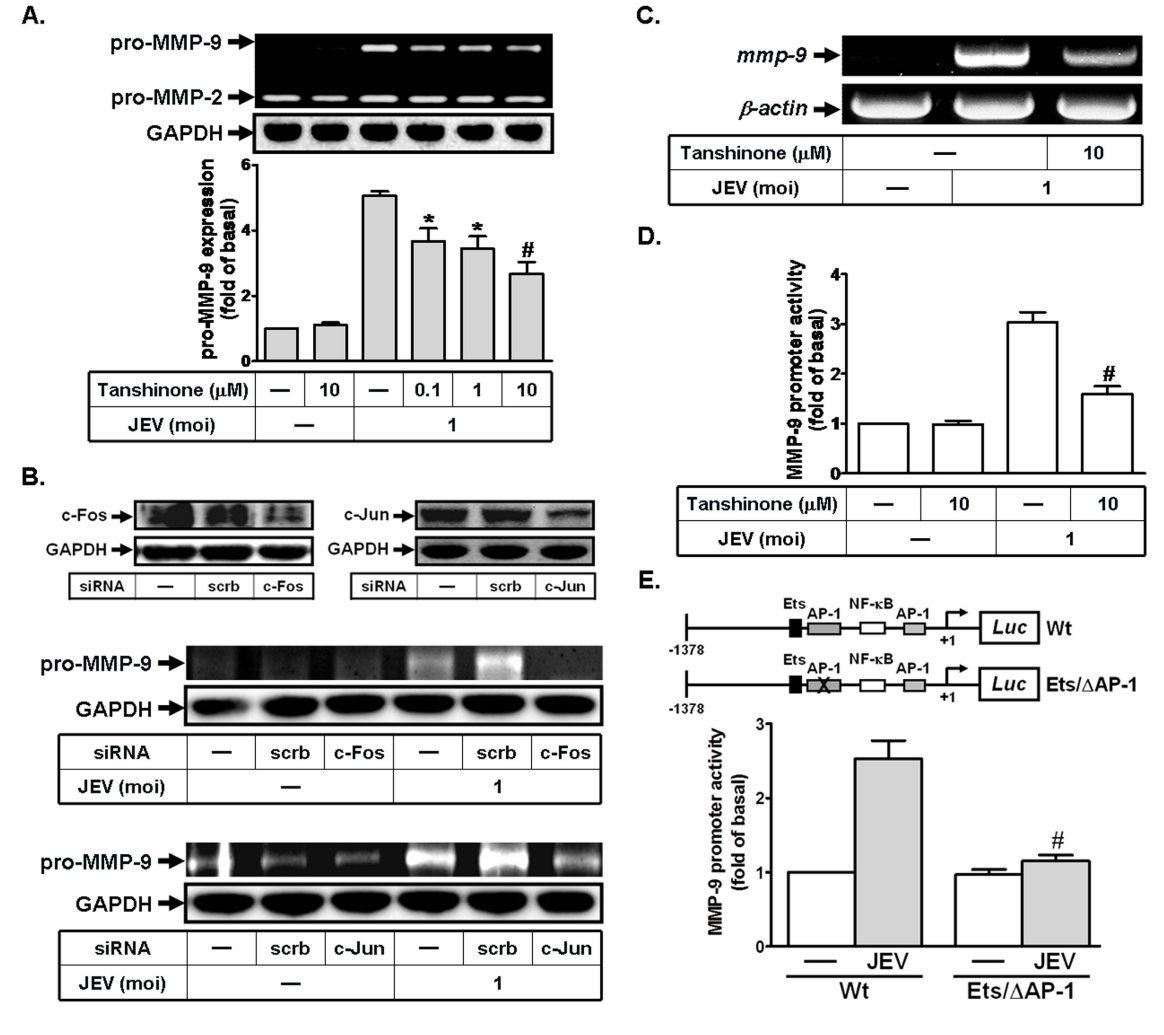
**Involvement of AP-1 in MMP-9 expression with JEV infection of RBA cells**. (A, B) Cells were pretreated with tanshinone for 1 h or transfected with siRNA of scrambled (scrb), c-Jun, or c-Fos, and then infected with JEV for 16 h. The conditioned media were used to determine MMP-9 expression by gelatin zymography. Cell lysates were analyzed by western blot using an anti-c-Jun, anti-c-Fos, or anti-GAPDH antibody. (C) Cells were pretreated with tanshinone for 1 h, and then incubated with JEV for 6 h. RNA samples were analyzed by RT-PCR to assess the levels of MMP-9 mRNA expression. (D, E) Cells were transiently transfected with MMP-9-luc reporter gene, pretreated with tanshinone (10 μM) for 1 h, and then incubated with JEV for 6 h. In addition, cells were transfected with wild-type MMP-9 promoter and AP-1-mutated MMP-9 promoter, and then incubated with JEV (moi = 1) for 6 h. MMP-9 promoter activity was determined in the cell lysates. Data are expressed as mean ± S.E.M. for five independent experiments. *P < 0.05; ^#^P < 0.01, as compared with the cells exposed to JEV alone (A, D). ^#^P < 0.01, as compared with cells transfected with wild-type MMP-9 promoter stimulated by JEV (E).

The promoter region of MMP-9 possesses an AP-1 binding site that is regulated by several external stimuli in different cell types [18-20]. Therefore, we first determined whether JEV-induced MMP-9 expression was mediated through AP-1 in RBA-1 cells. As shown by the gelatin zymographic experiments in Figure 1A, pretreatment with an inhibitor of AP-1 (tanshinone) attenuated JEV-induced MMP-9 expression in a concentration-dependent manner. Within the AP-1 subfamily, c-Jun is an important transcriptional activator and c-Fos transactivates MMPs by binding directly to promoter AP-1 motifs [21,28]. Thus, we used the siRNA transfection technique to verify whether c-Jun and c-Fos were required for MMP-9 expression induced by JEV. As shown in Figure 1B, transfection with either c-Jun or c-Fos siRNA down-regulated total c-Jun or c-Fos protein expression and significantly reduced JEV-induced MMP-9 expression in RBA cells.

Next, we found that the action of AP-1 in regulating MMP-9 expression occurred at the transcriptional level in RBA-1 cells, since pretreatment with tanshinone significantly attenuated JEV-induced MMP-9 mRNA accumulation (Figure 1C). To ensure the transcriptional regulation of MMP-9 gene in this context, RBA-1 cells were transfected with a luciferase reporter vector containing an exogenous MMP-9 promoter, and the cells were then stimulated with JEV for 6 h. As shown in Figure 1D, JEV infection stimulated MMP-9 promoter activity, which was attenuated by pretreatment with tanshinone in RBA cells. To further confirm the role of AP-1 in JEV-mediated MMP-9 promoter induction, a point-mutated AP-1 MMP-9 promoter construct was used. As shown in Figure 1E, JEV-stimulated MMP-9 promoter activity was prominently lost in RBA-1 cells transfected with the point-mutated AP-1 MMP-9 promoter. These results suggest that AP-1 (c-Jun and c-Fos) is required for JEV-induced MMP-9 expression in RBA-1 cells.

### AP-1 expression is mediated via c-Src, PDGFR, and PI3K/Akt by JEV infection

**Figure 2:**
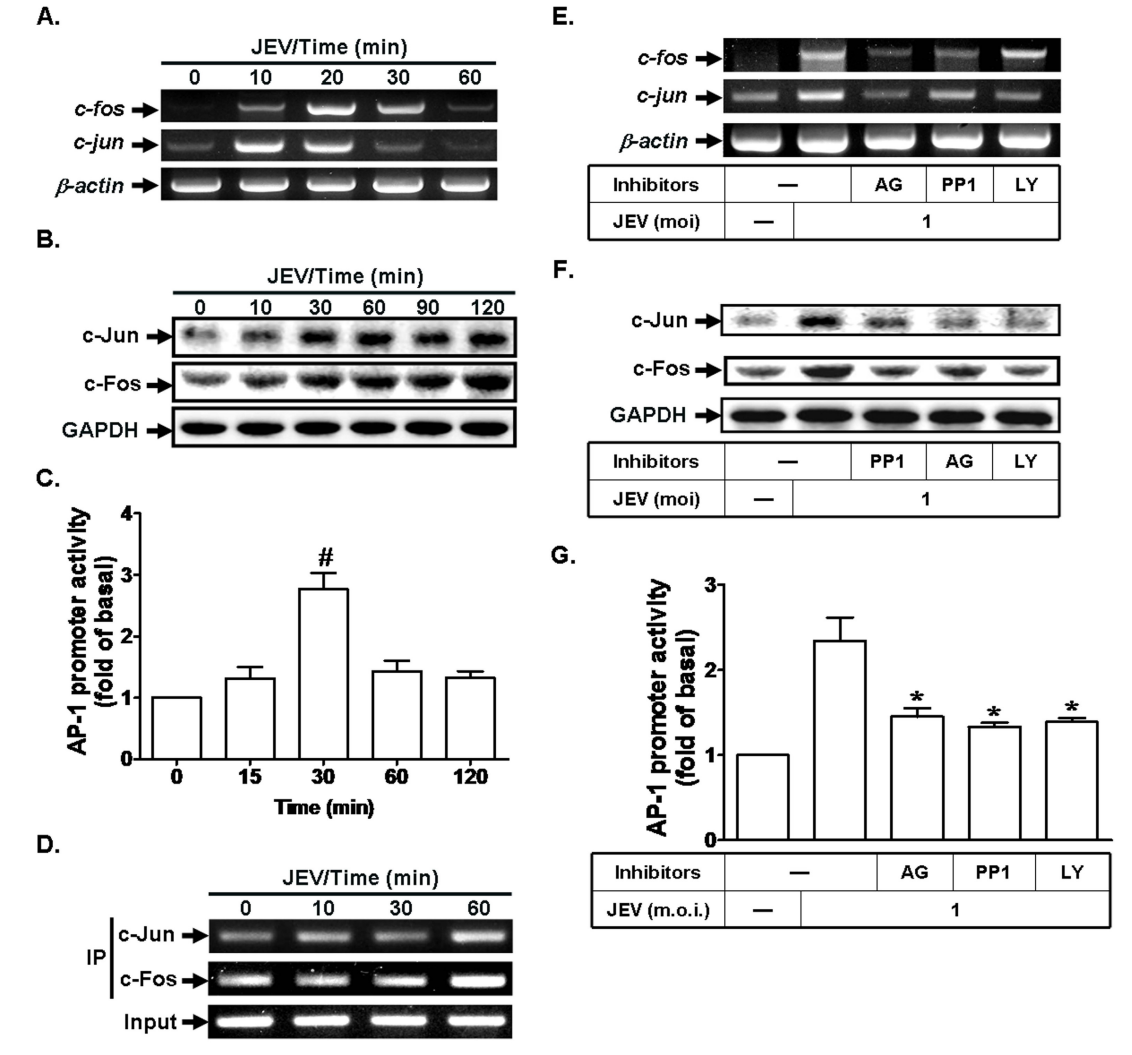
**EV71 infection induces activation of AP-1 via c-Src, PDGFR, and PI3K/Akt**. Cells were incubated with JEV (moi = 1) for the indicated time intervals. (A) mRNA levels for c-Jun and c-Fos were determined by RT-PCR. (B) Protein levels of c-Jun and c-Fos were determined by western blotting. (C) Cells were transfected with an AP-1 promoter luciferase construct or control vector (pGL3-Luc) together with a β-galactosidase plasmid, and then incubated with JEV (moi = 1) for the indicated time intervals. AP-1 promoter activity was normalized to that of β-galactosidase activity. (D) Cells were incubated with JEV (moi = 1) for the indicated time intervals. c-Fos and c-Jun binding activities were analyzed by chromatin immunoprecipitation (ChIP) assay. (E, F) Cells were pretreated with AG1296 (AG, 10 μM), PP1 (10 μM), or LY294002 (LY, 30 μM) for 1 h, and then incubated with JEV (moi = 1) for (E) 20 min or (F) 60 min. (E) mRNA expression for c-Jun and c-Fos were examined by RT-PCR. (F) Protein levels of c-Jun and c-Fos were determined by western blotting. (G) Cells were transfected with an AP-1 promoter luciferase construct or control vector (pGL3-Luc) together with a β-galactosidase plasmid, pretreated with AG1296 (AG, 10 μM), PP1 (10 μM), or LY294002 (LY, 30 μM) for 1 h, and then incubated with JEV (moi = 1) for 30 min. The AP-1 promoter activity was normalized to that of β-galactosidase activity. Data are expressed as mean ± S.E.M. for five independent experiments. ^#^P < 0.01, as compared with the basal level (C). *P < 0.05, as compared with cells exposed to JEV alone (G).

The regulation of AP-1 activity depends on changes in c-Jun and c-Fos gene transcription and mRNA accumulation [21]. In addition, we demonstrated that transfection of c-Jun or c-Fos siRNA diminished JEV-induced MMP-9 expression (Figure 1B). Therefore, we explored the up-regulation of AP-1 by JEV with respect to c-Jun and c-Fos mRNA expression, using RT-PCR. The results show that stimulation of RBA-1 cells with JEV induces c-Jun and c-Fos gene expression in a time-dependent manner. The expression of c-Jun and c-Fos by JEV infection reached a peak within 20 min and declined to basal levels within 60 min (Figure 2A). In addition, JEV also induced c-Jun and c-Fos protein expression in a time-dependent manner (Figure 2B). To further determine whether AP-1 transcriptional activity is regulated by JEV infection, RBA-1 cells were transfected with an AP-1-luciferase reporter gene. JEV infection enhanced AP-1 transcriptional activity in a time-dependent manner with a maximal response within 30 min (Figure 2C). These results indicate that JEV infection induces AP-1 activation through c-Jun and c-Fos in RBA-1 cells. On the other hand, we used a ChIP assay to determine whether JEV-stimulated recruitment of AP-1 to MMP-9 promoter is involved in MMP-9 gene expression. We designed a pair of primers for MMP-9 promoter (-597 to -318) region, containing an AP-1 binding site. Chromatin was immunoprecipitated using an anti-c-Fos or anti-c-Jun antibody, and the MMP-9 promoter region (-597 to -318) was amplified by PCR. As shown in Figure 2D, JEV stimulated in vivo binding of c-Fos and c-Jun to the MMP-9 promoter in a time-dependent manner with a maximal response within 60 min.

Previous studies have reported that AP-1 activation is mediated through PDGFR signaling pathways [22,23]. In addition, our previous study reported that enterovirus 71 (EV71) induces AP-1 activation via a c-Src/PDGFR/PI3K/Akt cascade in RBA-1 cells [29]. Therefore, to further determine whether c-Jun/c-Fos gene expression and AP-1 transcriptional activity are mediated through activation of c-Src, PDGFR, and PI3K/Akt by JEV infection, inhibitors of PDGFR (AG1296), c-Src (PP1), or PI3K/Akt (LY294002) were used to assess transcriptional activity. These results show that JEV-enhanced c-Jun/c-Fos protein levels, mRNA expression, and AP-1 transcriptional activity were significantly attenuated by pretreatment with AG1296, PP1, or LY294002 (Figures 2E-G). These results suggest that JEV-stimulated AP-1 activation is mediated through c-Src, PDGFR, and PI3K/Akt in RBA-1 cells.

### JEV-induced proMMP-9 expression is mediated via a c-Src/PDGFR signaling

**Figure 3:**
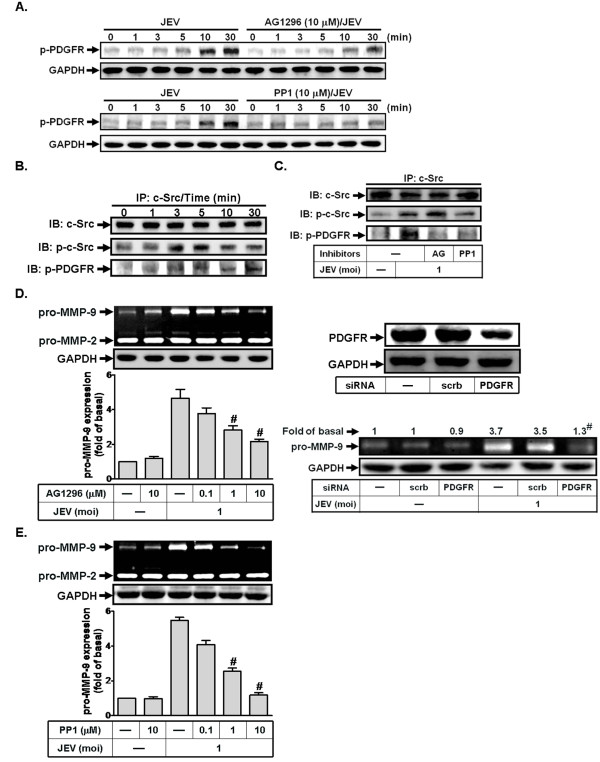
**JEV infection induces MMP-9 expression through c-Src/PDGFR in RBA-1 cells**. (A) Cells were pretreated with AG1296 (10 μM) or PP1 (10 μM) for 1 h, and then infected with JEV (moi = 1) for the indicated time intervals. The cell lysates were analyzed by western blot using an anti-phospho-PDGFR or anti-GAPDH antibody. (B, C) Cells were pretreated without or with AG1296 (AG, 10 μM) or PP1 (10 μM) for 1 h, and then incubated with JEV (moi = 1) for the indicated time intervals (B) or 5 min (C). Cell lysates were subjected to immunoprecipitation using an anti-c-Src antibody. The immunoprecipitates were analyzed by western blot using an anti-c-Src, anti-phospho-c-Src, or anti-phospho-PDGFR antibody. (D, E) Cells were pretreated with AG1296 or PP1 for 1 h or transfected with PDGFR siRNA, followed by incubation with JEV for 16 h. Cell lysates were analyzed by western blot using an anti-PDGFR or anti-GAPDH antibody. The conditioned media were used to determine MMP-9 expression by gelatin zymography. Data are expressed as mean ± S.E.M. for five independent experiments. ^#^P < 0.01, as compared with cells exposed to JEV alone [D (left panel), E]. ^#^P < 0.01, as compared with cells transfected with scrambled siRNA exposed to JEV (D, right panel).

To determine if PDGFR activation occurs upon exposure of JEV, phosphorylated PDGFR was determined by western blot using specific antibody to the active form of PDGFR. As shown in Figure 3A, JEV infection stimulated PDGFR phosphorylation in a time-dependent manner, which was inhibited by pretreatment with AG1296 (an inhibitor of PDGFR). Previous studies have reported that growth factor receptors are activated through transactivation of activated c-Src by various stimuli [30]. Therefore, we determined whether c-Src mediates transactivation of PDGFR in response to JEV infection. As depicted in Figure 3A, JEV-stimulated PDGFR phopsphorylation was reduced by pretreatment with PP1 (an inhibitor of c-Src). Moreover, a co-immunoprecipitation study revealed that JEV infection-stimulated c-Src directly associated with PDGFR in a time-dependent manner with a maximal response within 3-5 min (Figure 3B). Further, pretreatment with PP1, but not AG 1296, diminished JEV infection-induced c-Src phosphorylation (Figure 3C). These results indicate that c-Src is an upstream component of PDGFR in JEV-mediated responses in RBA-1 cells.

We further determined whether JEV-induced MMP-9 expression is mediated through c-Src/PDGFR in RBA-1 cells. As shown in Figures 3D and 3E, pretreatment with either AG1296 or PP1 attenuated JEV-induced MMP-9 expression in a concentration-dependent manner. Further, transfection of PDGFR siRNA attenuated JEV-induced MMP-9 expression in RBA-1 cells (Figure 3D). All these results together suggest that JEV-induced MMP-9 expression is mediated through the c-Src/PDGFR/AP-1 cascade in RBA-1 cells.

### Involvement of PI3K/Akt pathway in JEV-induced proMMP-9 expression

**Figure 4:**
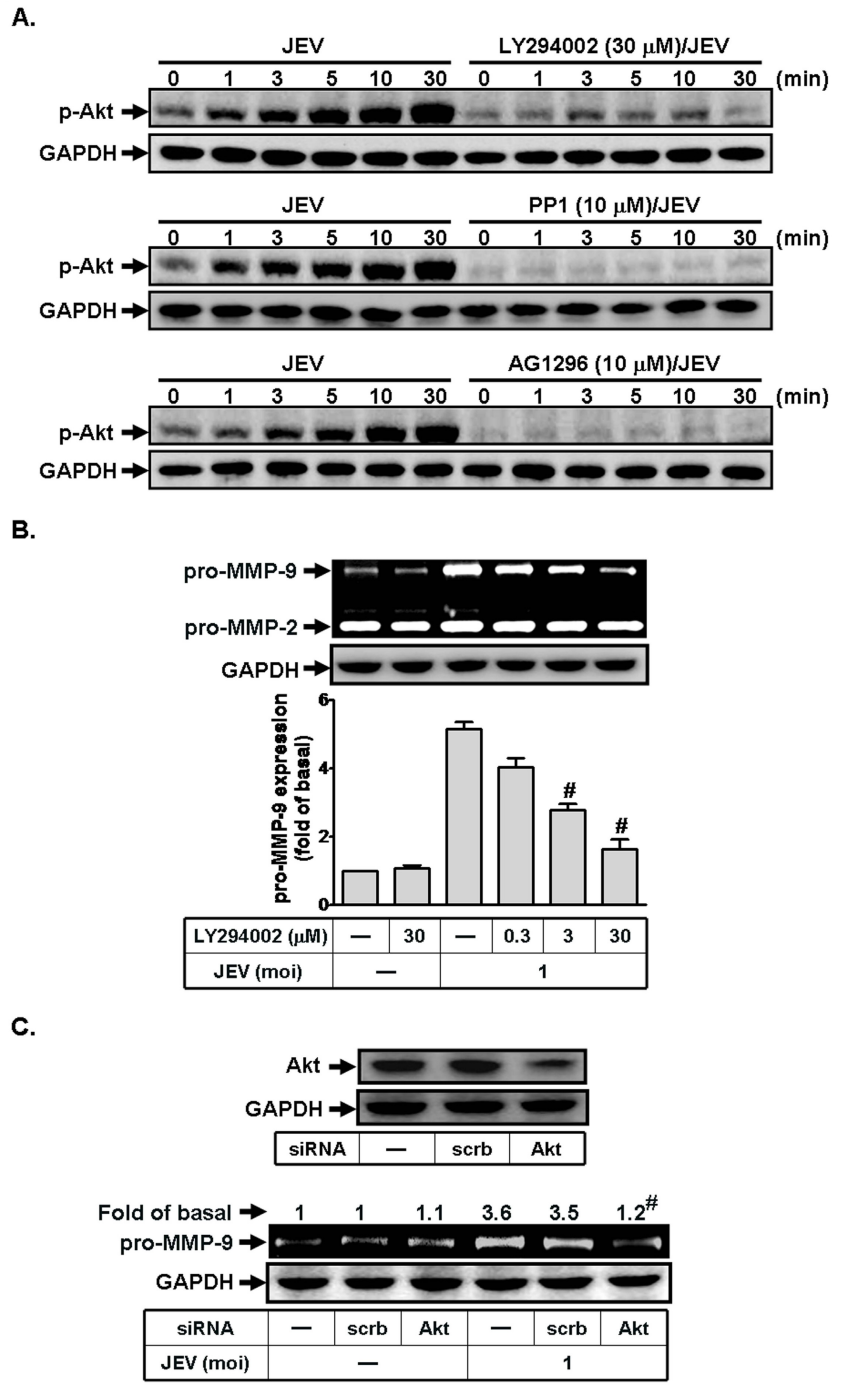
**Involvement of PI3K/Akt in JEV-induced MMP-9 expression in RBA-1 cells**. (A) Cells were pretreated with LY294002 (30 μM), AG1296 (10 μM), or PP1 (10 μM) for 1 h, and then infected with JEV (moi = 1) for the indicated time intervals. Cell lysates were analyzed by western blot using an anti-phospho-Akt or anti-GAPDH antibody. (B, C) Cells were pretreated with LY294002 for 1 h or transfected with Akt siRNA, followed by incubation with JEV for 16 h. The cell lysates were analyzed by western blot using an anti-Akt or anti-GAPDH antibody. The conditioned media were used to determine MMP-9 expression by gelatin zymography. Data are expressed as mean ± S.E.M. for five independent experiments. ^#^P < 0.01, as compared with the cells exposed to JEV alone.

Next, we investigated whether JEV-induced MMP-9 expression is mediated through PI3K/Akt signaling in RBA-1 cells. First, we verified that Akt is activated upon exposure to JEV, using an antibody specific for the phosphorylated, active form of Akt, by western blotting. As shown in Figure 4A, JEV infection-increased Akt phosphorylation was observed in a time-dependent manner with a maximal response within 5 min, which was inhibited by pretreatment with LY294002 during the period of observation (Figure 4A). In addition, it is known that PI3K/Akt is activated following stimulation of receptor tyrosine kinases by different stimuli in various cell types [30-33]. Therefore, we used AG1296 and PP1 to confirm this possibility in this cascade. As illustrated in Figure 4A, JEV-stimulated Akt phosphorylation was attenuated by pretreatment with either AG1296 or PP1, indicating that JEV infection-stimulated PI3K/Akt activation was mediated through c-Src/PDGFR in RBA-1 cells.

We further determined whether JEV-induced MMP-9 expression is mediated through PI3K/Akt in RBA-1 cells. As shown in Figures 4B and 4C, pretreatment with LY294002 or transfection with Akt siRNA attenuated JEV-induced MMP-9 expression in RBA-1 cells. Taken together, these results suggest that JEV-induced MMP-9 expression is mediated through c-Src/PDGFR/PI3K/Akt/AP-1 signaling in RBA-1 cells.

### c-Src, PDGFR, and PI3K/Akt are required for JEV-induced MMP-9 mRNA expression

**Figure 5:**
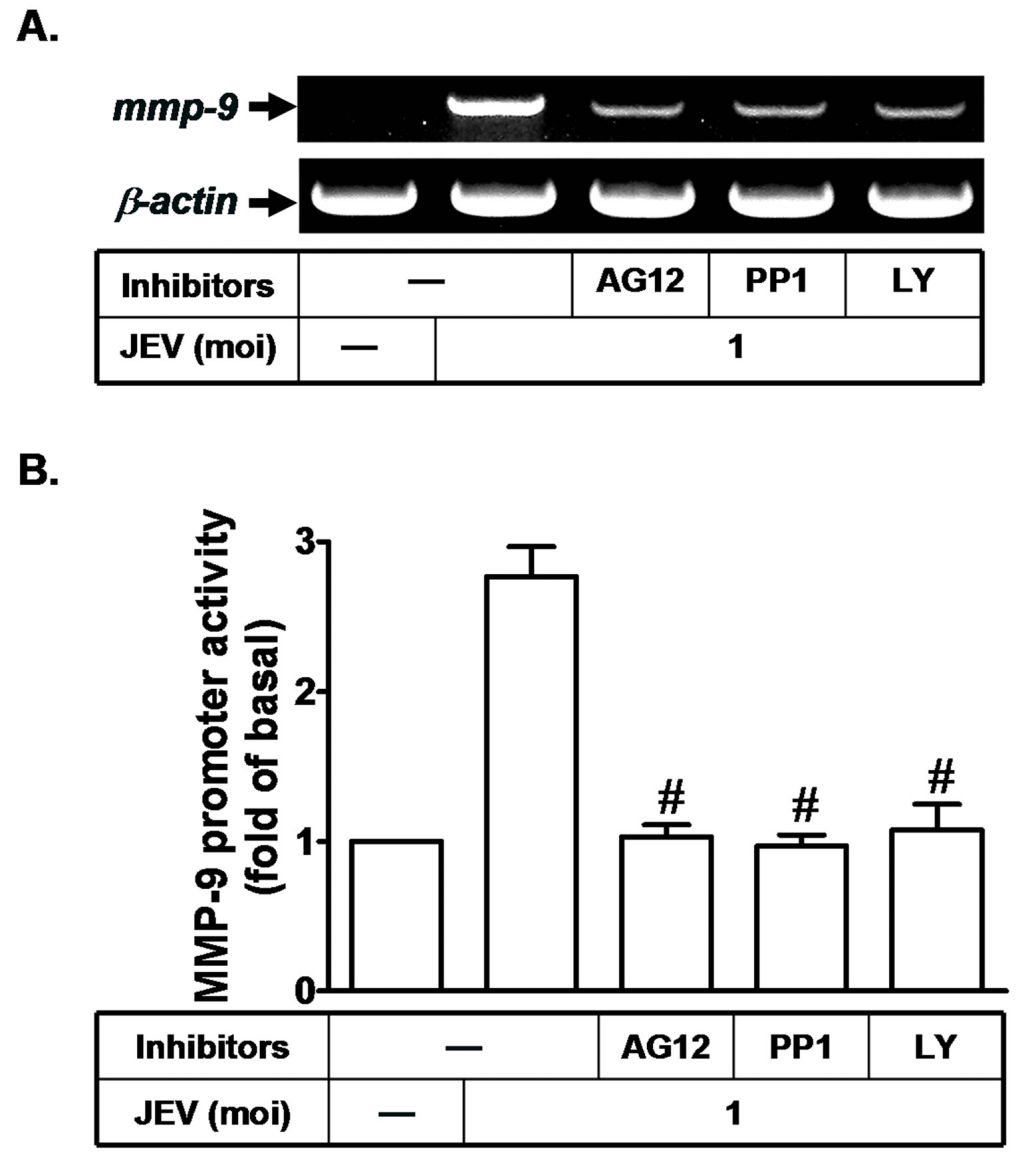
**Involvement of c-Src/PDGFR/PI3K/Akt in MMP-9 mRNA expression induced by JEV in RBA-1 cells**. (A) Cells were pretreated with AG1296 (AG12, 10 μM), PP1 (10 μM), or LY294002 (LY, 30 μM) for 1 h, and then infected with JEV for 6 h. RNA samples were analyzed by RT-PCR to determine levels of MMP-9 mRNA. (B) Cells were transiently transfected with an MMP-9-luc reporter gene, pretreated with AG1296 (AG12, 10 μM), PP1 (10 μM), or LY294002 (LY, 30 μM) for 1 h, and then infected with JEV for 6 h. The promoter activity of MMP-9 was measured. Data are expressed as mean ± S.E.M. for five independent experiments. ^#^P < 0.01, as compared with the cells exposed to JEV alone.

We further examined whether c-Src, PDGFR, and Akt are involved in regulation of MMP-9 expression at the transcriptional level in RBA-1 cells. As shown in Figures 5A and 5B, pretreatment of RBA cells with AG1296, LY294002, or PP1 significantly attenuated JEV-induced MMP-9 mRNA accumulation and MMP-9 promoter activity. These results further confirm that in JEV-infected RBA-1, up-regulation of MMP-9 gene through activation of c-Src, PDGFR, and Akt mainly occurrs at the transcriptional level.

### JEV-induced AP-1 expression is mediated via MAPKs

**Figure 6:**
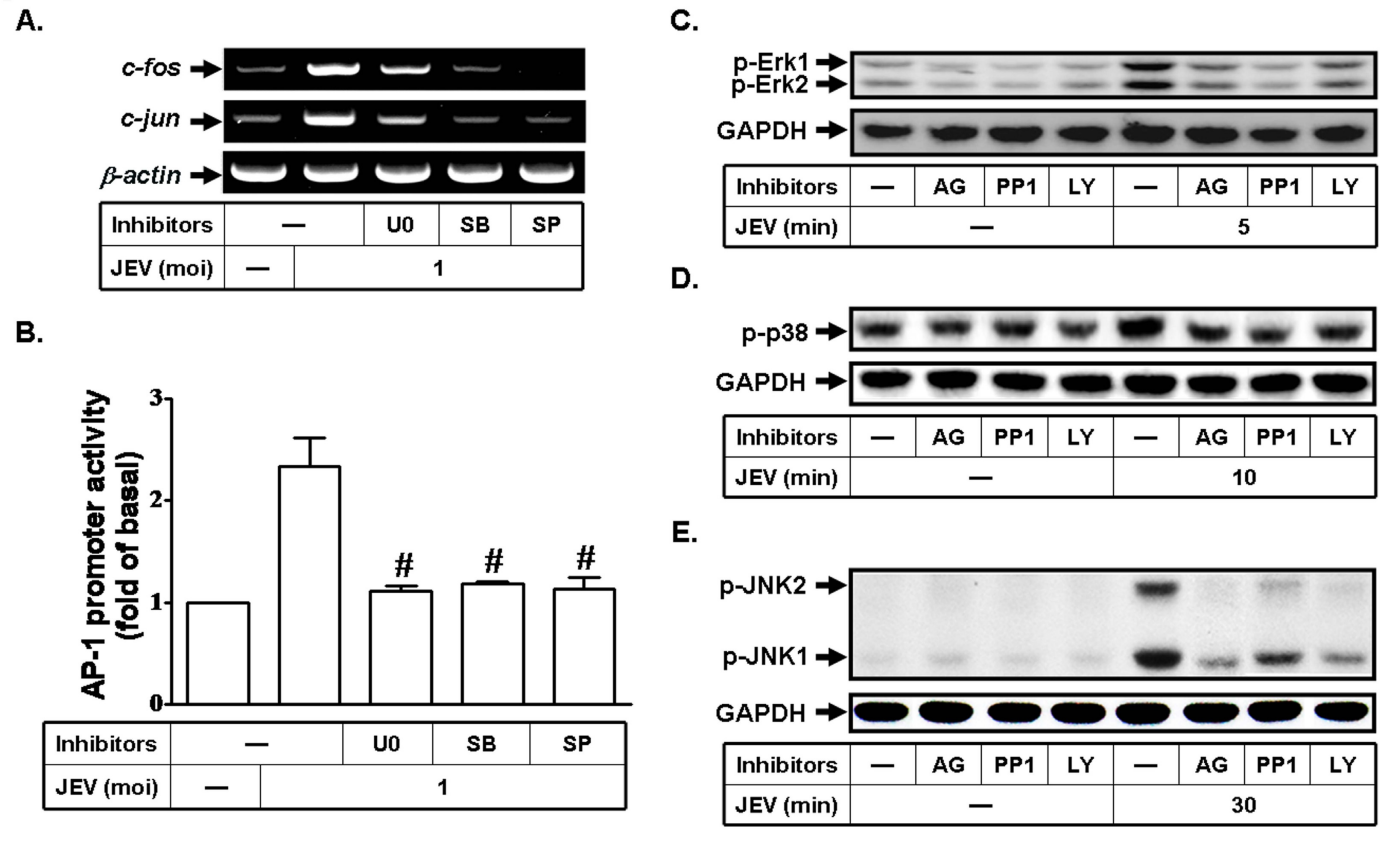
**JEV infection activates AP-1 via c-Src/PDGFR/PI3K/Akt/MAPKs pathway**. (A) Cells were pretreated with U0126 (U0, 1 μM), SB203580 (SB, 1 μM), or SP600125 (SP, 1 μM) for 1 h, followed by stimulation with JEV for 20 min. The isolated RNA samples were analyzed by RT-PCR, using the primers specific for c-Fos, c-Jun, and β-actin. (B) Cells were transfected with an AP-1 promoter luciferase construct together with a β-galactosidase plasmid, pretreated with U0126 (U0, 1 μM), SB203580 (SB, 1 μM), or SP600125 (SP, 1 μM) for 1 h, and then incubated with JEV for 30 min. AP-1 promoter activity was normalized to that of β-galactosidase activity. (C-E) Cells were pretreated with AG1296 (AG, 10 μM), PP1 (10 μM), or LY294002 (LY, 30 μM) for 1 h, and then infected with JEV (moi = 1) for (C) 5, (D) 10, or (E) 30 min. The cell lysates were analyzed by western blot using an anti-phospho-p42/p44 MAPK, anti-phospho-p38 MAPK, anti-phospho-JNK1/2, or anti-GAPDH antibody. Data are expressed as mean ± S.E.M. for five independent experiments. ^#^P < 0.01, as compared with the cells exposed to JEV alone.

Our previous study has shown that the mechanism whereby JEV infection leads to the expression of MMP-9 is mediated via p42/p44 MAPK, p38 MAPK, and JNK1/2 in RBA-1 cells [15]. In addition, previous studies have reported that AP-1 activation is also mediated through MAPKs signaling stimulated by various factors in various cell types [21,28]. Thus, we investigated whether activation of AP-1 by JEV infection is mediated through MAPKs signaling in RBA-1 cells. AP-1 activation was assessed following JEV infection in the presence of inhibitor for p42/p44 MAPK (U0126), p38 MAPK (SB203580), or JNK1/2 (SP600125). These data show that JEV-induced c-Jun/c-Fos gene expression and AP-1 transcriptional activity were significantly blocked by pretreatment with U0126, SB203580, and SP600125 (Figures 6A and 6B). These results suggest that JEV-induced AP-1 activation is MAPKs dependent in RBA-1 cells.

The receptors for epidermal growth factor (EGF), PDGF, brain-derived neurotrophic factor (BDNF), and nerve growth factor (NGF) regulate many signaling components involved in MAPKs cascades [34]. Thus, we determined whether activation of MAPKs by JEV infection in RBA-1 cells is mediated through c-Src/PDGFR/PI3K/Akt pathway. Phosphorylation of p42/p44 MAPK, p38 MAPK, and JNK1/2 by JEV infection was decreased by pretreatment with AG1296, PP1, and LY294002 in RBA-1 cells (Figures 6C-E). Taken together, these results suggest that c-Src/PDGFR/PI3K/Akt/MAPKs/AP-1 signaling is involved in MMP-9 expression induced by JEV infection in RBA-1 cells.

### c-Src/PDGFR/PI3K/Akt cascade activation is dependent on ROS by JEV infection

**Figure 7:**
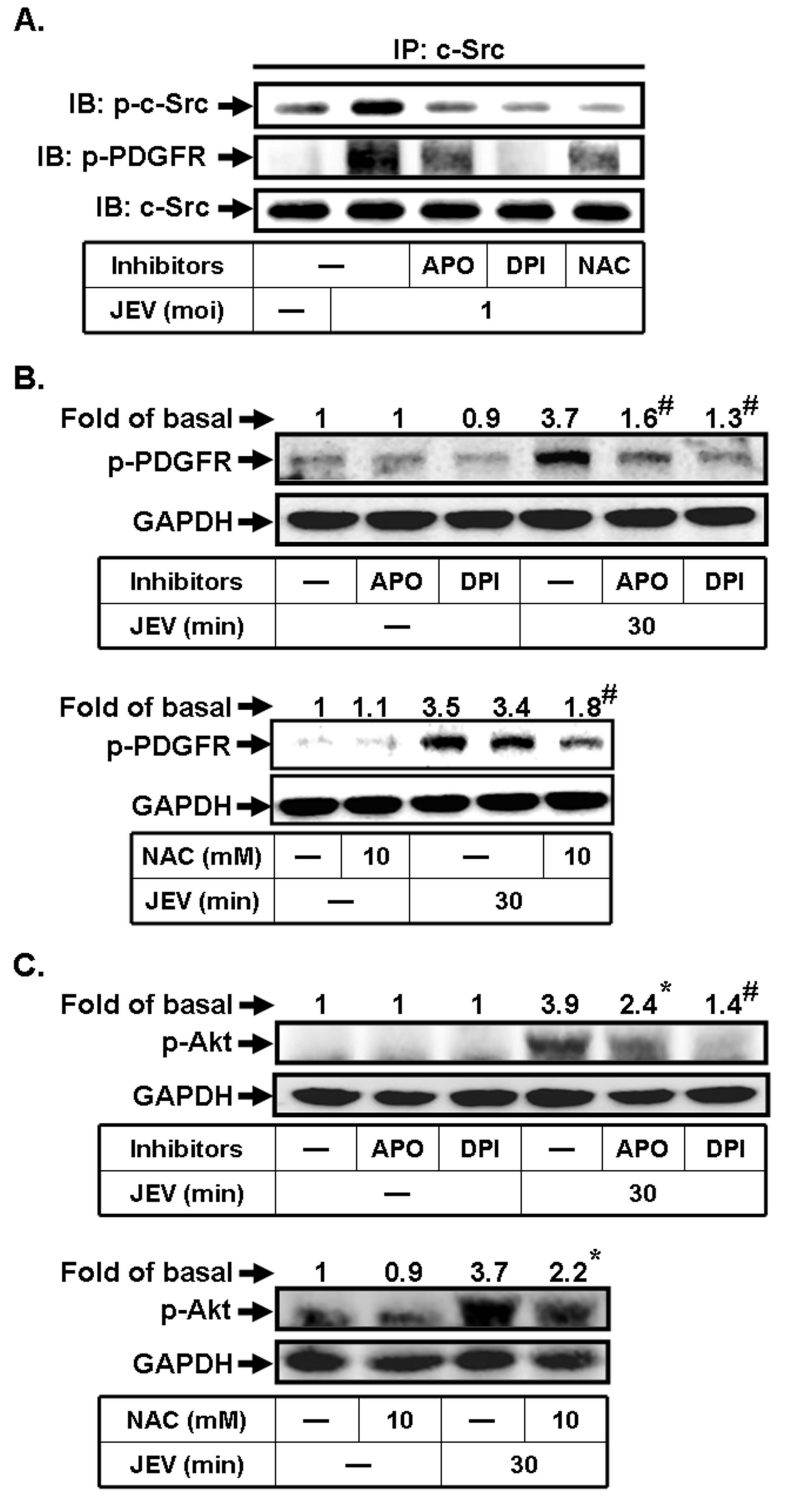
**JEV-induced c-Src/PDGFR/PI3K/Akt is mediated via ROS in RBA-1 cells**. (A) Cells were pretreated with APO (100 μM), DPI (10 μM), or NAC (10 mM) for 1 h, and then stimulated with JEV for 5 min. The cell lysates were subjected to immunoprecipitation using an anti-c-Src antibody. The immunoprecipitates were analyzed by western blot analysis using an anti-c-Src, anti-phospho-c-Src, or anti-phospho-PDGFR antibody. (B, C) Cells were pretreated with APO (100 μM), DPI (10 μM), or NAC (10 mM) for 1 h, and then incubated with JEV for 30 min. The cell lysates were analyzed by western blot using an anti-phospho-PDGFR, anti-phospho-Akt, or anti-GAPDH antibody. Data are expressed as mean ± S.E.M. for five independent experiments. *P < 0.05; ^#^P < 0.01, as compared with the cells exposed to JEV alone.

Our previous study has shown that JEV infection induces ROS generation through NADPH oxidase, which in turn activates MAPKs pathway in RBA-1 cells [15]. Moreover, several studies indicate that ROS production leads to c-Src activation, which strongly increases kinase activity [35-37]. Therefore, to investigate whether activation of c-Src by JEV infection is mediated through ROS, inhibitors of NADPH oxidase (APO and DPI) and a ROS scavenger (NAC) were used. As shown in Figure 7A, pretreatment with APO, DPI, or NAC attenuated JEV-stimulated PDGFR and c-Src phosphorylation in the complex immunoprecipitated by using an anti-c-Src antibody, indicating that JEV-stimulated c-Src/PDGFR activation is mediated through NADPH oxidase/ROS generation in RBA-1 cells. Next, we determined whether NADPH oxidase/ROS modulates PDGFR and PI3K/Akt signaling pathway by JEV infection in RBA-1 cells. As shown in Figures 7B and 7C, pretreatment with APO, DPI, or NAC significantly attenuated JEV-stimulated PDGFR and Akt phosphorylation. Taken together, these results indicate that JEV-stimulated phosphorylation of c-Src/PDGFR/PI3K/Akt pathway is mediated through NADPH oxidase/ROS in RBA-1 cells.

## Discussion

Neurotropic viruses can cause massive neuronal dysfunction and destruction that leads to neurological diseases [1]. Based on neural cell composition and the barrier between the peripheral tissues and CNS, astrocytes might play a role in the transmission of virus from peripheral blood flow into the CNS. Recent studies have demonstrated a relationship between elevated levels of MMP-9 and severity of several pathological states in the CNS [5,6]. MMP-9 has been shown to degrade components of the basal lamina, leading to disruption of the BBB, and to contribute to neuroinflammatory responses in many neurological diseases [38]. Several lines of evidence have shown that reduction of MMP activity by pharmacological inhibitors or gene knock-out strategies protects the brain from BBB disruption, cell death, and advanced neuroinflammation [5,39]. Previous studies have indicated that several signaling cascades are involved in MMP-9 expression by virus infection [13,14]. We have previously demonstrated that JEV infection induces MMP-9 expression via NF-κB in RBA-1 cells [15]. Moreover, AP-1 is also known to play an important role in MMP-9 expression in various cell types [18]. However, little is known about the molecular mechanisms of JEV-induced AP-1 activation leading to MMP-9 expression in RBA-1 cells. In this study, the mechanisms underlying JEV-induced MMP-9 expression were investigated using selective pharmacological inhibitors or transfection with siRNAs. The requirement of transcription factors for the regulation of JEV-induced MMP-9 gene expression was determined by reporter gene assays. These results demonstrate that JEV induces MMP-9 expression via a ROS, c-Src, PDGFR, PI3K/Akt, p42/p44 MAPK, p38 MAPK, and JNK1/2-dependent pathway following activation of transcription factor AP-1 (c-Jun and c-Fos) in RBA-1 cells.

Previous studies have reported that the promoter of MMP-9 possesses a series of functional activator element-binding sites, including NF-κB and AP-1 [6,9]. In addition, AP-1 activity is enhanced by various factors including growth factors, cytokines, physical and chemical stresses, and bacterial and viral infections [21,28]. However, AP-1 participation in MMP-9 expression is poorly understood in JEV-infected RBA-1 cells. First, we therefore determined the requirement for AP-1 in JEV-induced MMP-9 expression. Our results reveal that JEV infection stimulates expression of MMP-9, which was significantly inhibited by pretreatment with tanshinone and transfection with c-Jun siRNA and c-Fos siRNA. In addition, JEV-induced MMP-9 mRNA expression and promoter activity were attenuated by pretreatment with tanshinone or transfection with a point-mutated AP-1 MMP-9 promoter, indicating that AP-1 participates in MMP-9 expression by JEV infection in RBA-1 cells. Moreover, we demonstrated that JEV-induced AP-1 activation occurs through changes in c-Jun and c-Fos gene transcription and mRNA turnover. These results are consistent with previous studies demonstrating that enhanced expression of MMP-9 in Epstein-Barr virus (EBV)-infected or HBV-infected cells is mediated through activation of AP-1 transcriptional activity [40,41].

Several factors enhance AP-1 activity through activation of many signaling pathways, such as PDGF-induced activation of AP-1 through p42/p44 MAPK and JNK1/2 in NIH 3T3 mouse fibroblasts [22,23]. In addition, our previous study reported that EV71 induces AP-1 activation via a c-Src/PDGFR/PI3K/Akt cascade in RBA-1 cells [29]. However, activation of c-Src, PDGFR, and PI3K/Akt by JEV is poorly understood in RBA-1 cells. Therefore, our results from this present study reveal that JEV infection induces expression of c-Jun and c-Fos, and that these expressions are significantly inhibited by pretreatment with AG1296, PP1, or LY294002. In accord with our recent findings of COX-2 expression with EV71 infection in RBA-1 cells [29], these data suggest that AP-1 activation by JEV infection is mediated through a c-Src, PDGFR, and PI3K/Akt pathway.

Next, we investigated the roles of c-Src, PDGFR, and PI3K/Akt in MMP-9 expression in RBA-1 cells. Our results show that JEV infection stimulates phosphorylation of PDGFR, which is attenuated by pretreatment with AG1296 and PP1. In addition, co-immunoprecipitation assays were performed to ensure that protein levels of p-PDGFR and p-c-Src time-dependently increase in a c-Src-immunoprecipitated complex stimulated by JEV infection, which was inhibited by pretreatment with AG1296 or PP1. Moreover, several studies have reported that Akt is activated following stimulation of receptor tyrosine kinase by different stimuli [30-32]. In addition, in rat brain astrocyte cells or neural cells, PI3K/Akt activation has been shown to be mediated through PDGFR transactivation [42-45]. In this study, pretreatment of RBA-1 cells with AG1296 or PP1 inhibited JEV-stimulated Akt phosphorylation, indicating that activation of PDGFR and c-Src are required for this response. Apart from these, pretreatment with AG1296, PP1, or LY294002; or transfection with siRNA of PDGFR or Akt significantly inhibited JEV-induced MMP-9 protein expression and mRNA accumulation. These data indicate that PI3K/Akt activation is mediated through c-Src-dependent transactivation of PDGFR, which promotes AP-1 activation and eventually leads to MMP-9 expression with JEV infection of RBA-1 cells. This result is consistent with recent studies reporting that MMP-9 expression induced by IL-1β is mediated via activation of c-Src/PDGFR/PI3K/Akt in various cell types [45,46].

Previous studies have shown that AP-1 activation is also mediated through MAPKs signaling pathways by various factors in various cell types [21]. In addition, our previous study has shown that JEV infection-induced MMP-9 expression is mediated via ROS-p42/p44 MAPK, p38 MAPK, and JNK1/2 in RBA-1 cells [15]. Thus, we also investigated the roles of MAPKs in JEV-induced AP-1 activation. Our results reveal that JEV infection induces expression of c-Jun and c-Fos, which are significantly inhibited by pretreatment with U0126, SP600125, or SB203580. These data indicate that JEV-induced AP-1 activation is dependent on MAPKs in RBA-1 cells. Moreover, the MAPKs signaling cascade can be activated by growth factors such as PDGF [34]. Therefore, we examined whether MAPKs activation by JEV infection is mediated through a c-Src/PDGFR/PI3K/Akt pathway. In this study, pretreatment with AG1296, PP1, or LY294002 inhibited JEV-stimulated phosphorylation of p42/p44 MAPK, p38 MAPK, and JNK1/2, indicating that activation of c-Src/PDGFR/PI3K/Akt pathway by JEV infection regulates MAPKs activation in RBA-1 cells. These results suggest that expression of MMP-9 with JEV infection is mediated through a c-Src/PDGFR/PI3K/Akt/MAPKs pathway, associated with activation of transcription factor AP-1 in RBA-1 cells. Next, we investigated the role of ROS in activation of a c-Src/PDGFR/PI3K/Akt pathway by JEV infection in RBA-1 cells. Our data reveal that JEV infection-stimulated phosphorylation of PDGFR, c-Src, and Akt are attenuated by pretreatment with APO, DPI, or NAC. These data suggest that ROS plays an important role in JEV-stimulated activation of the c-Src/PDGFR/PI3K/Akt pathway in RBA-1 cells. Although MMP-9 induction is mediated by various stimuli and signaling pathways, such as ROS/ERK1/2, JNK1/2/NF-κB, PKCd/ERK1/2/Elk-1, and Ras/Raf/MEK/ER1/2/NF-κB [27,47,48], our results are the first to show a novel role for a ROS-dependent c-Src/PDGFR/PI3K/Akt/MAPKs/AP-1 signaling pathway in JEV-induced MMP-9 expression in RBA-1 cells. In the future, we will investigate the detailed mechanisms underlying JEV-induced MMP-9 expression in RBA-1 cells.

## Conclusion

**Figure 8:**
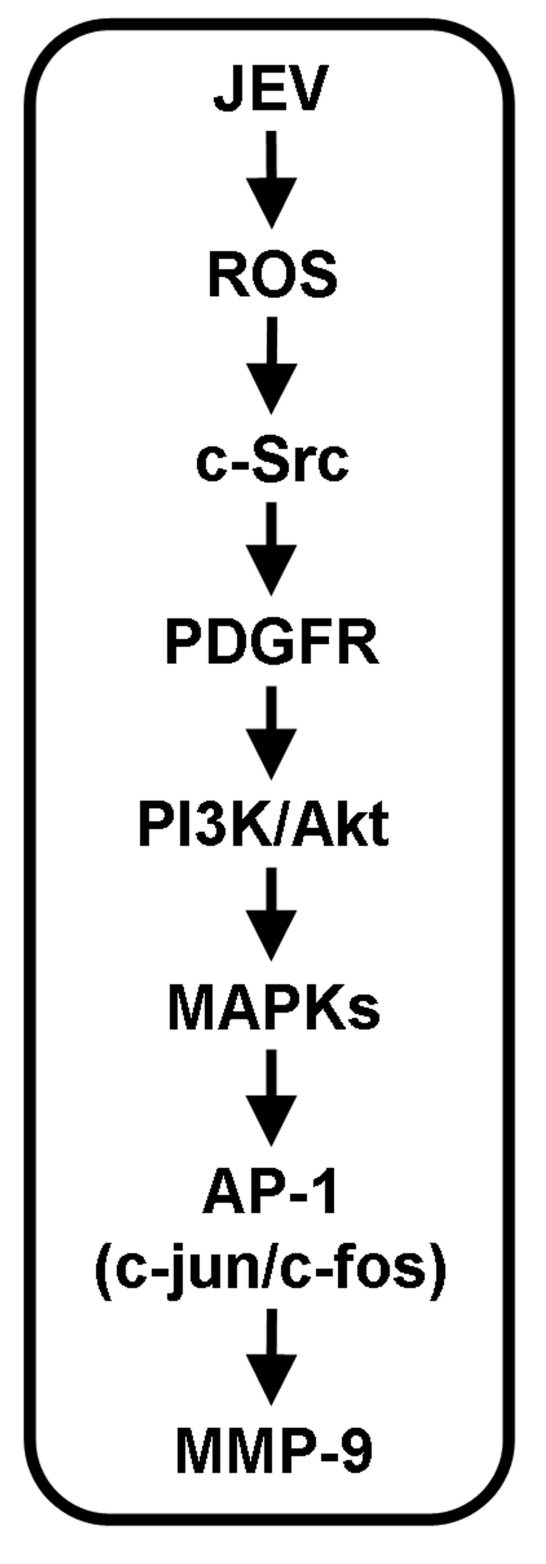
**Proposed model to illustrate the signaling pathways involved in MMP-9 expression in RBA-1 cells infected with JEV**. JEV-induced MMP-9 expression is mediated through ROS/c-Src/PDGFR/PI3K/Akt/MAPKs leading to AP-1 activation in RBA-1 cells.

In this study, we investigated alternative mechanisms underlying JEV-induced expression of MMP-9 in RBA-1 cells. This study demonstrates that JEV induces MMP-9 expression, which is mediated through a ROS-dependent c-Src/PDGFR/PI3K/Akt/MAPKs signaling pathway leading to immediate early gene AP-1 activation (c-Jun and c-Fos expression) in these cells. Based on observations from the literature and on our findings, Figure 8 depicts a model for the molecular mechanisms underlying JEV-induced MMP-9 expression in RBA-1 cells. These findings of JEV-induced MMP-9 expression in brain astrocytes imply that JEV might play a crucial role in the development of brain injuries and CNS diseases and provide useful support for the development of effective therapeutic targets in brain inflammation.

## Competing interests

The authors declare that they have no competing interests.

## Authors' contributions

CCL designed and performed experiments, acquisition and analysis of data, and drafted the manuscript. ITL, YHL and Caleb MY helped to perform experiments and prepare the manuscript. WJC and MJJ contributed to prepare JEV and RBA-1 cells. LDH performed experiments and prepared graphs. CMY has conceived of the study, participated in its design and coordination, has been involved in drafting the manuscript and revising it critically for important intellectual content and have given final approval of the version to be published. All authors have read and approved the final version of this manuscript.
